# Predictors of Discharge Against Medical Advice in a Tertiary Paediatric Hospital

**DOI:** 10.3390/ijerph16081326

**Published:** 2019-04-12

**Authors:** Louise Sealy, Karen Zwi, Gordon McDonald, Aldo Saavedra, Lisa Crawford, Hasantha Gunasekera

**Affiliations:** 1 Community Child Health, Sydney Children’s Hospital, Randwick, NSW 2031, Australia; louise.sealy@health.nsw.gov.au; 2 Sydney Informatics Hub, The University of Sydney, Sydney, NSW 2008, Australia; gordon.mcdonald@sydney.edu.au; 3 Centre for Translational Data Science; The University of Sydney, Sydney, NSW 2006, Australia; aldo.saavedra@sydney.edu.au; 4 Faculty of Health Sciences, The University of Sydney, Sydney, NSW 2006, Australia; 5 The Children’s Hospital at Westmead, Sydney, NSW 2145, Australia; dixie@sourcenation.com.au (L.C.); hasantha.gunasekera@health.nsw.gov.au (H.G.); 6 Children’s Hospital Westmead Clinical School, The University of Sydney, Sydney, NSW 2145, Australia

**Keywords:** discharge, self-discharge, treatment refusal, discharge against medical advice, discharge at own risk

## Abstract

Background: Patients who discharge against medical advice (DAMA) from hospital carry a significant risk of readmission and have increased rates of morbidity and mortality. We sought to identify the demographic and clinical characteristics of DAMA patients from a tertiary paediatric hospital. Methods: Data were extracted retrospectively from electronic medical records for all inpatient admissions over a 5-year period. Demographic characteristics (age, sex, Aboriginality, socioeconomic status and remoteness of residence) and clinical characteristics (admitting hospital site, level of urgency on admission, diagnosis and previous DAMA) were extracted and logistic regression models were used to identify predictors of DAMA with 95% confidence intervals. Results: There were 246,359 admissions for 124,757 patients, of which 1871 (0.8%) admissions and 1730 patients (1.4%) DAMA. Predictors of DAMA in a given admission were hospital site (OR 4.8, CI 4.2–5.7, *p* < 0.01), a mental health/behavioural diagnosis (OR 3.3, CI 2.2–4.8, *p* < 0.01), Aboriginality (OR 1.6, CI 1.3–2.1, *p* < 0.01), emergency rather than elective admissions (OR 0.7ha, CI 0.6–0.8, *p* < 0.01), a gastrointestinal diagnosis (OR 1.5, CI 1.1–2.0, *p* = 0.04) and a history of previous DAMA (OR 2.0, CI 1.2–3.2, *p* = 0.05). Conclusions: There are clear predictors of DAMA in this tertiary hospital admission cohort and identification of these provides opportunities for intervention at a practice and policy level in order to prevent adverse outcomes.

## 1. Introduction

Discharge against medical advice (DAMA) also known as “self-discharge” or “discharge at own risk” occurs when a patient (or in the case of a young child, their parent or carer) leaves hospital before their medical team recommends discharge. Worldwide, adult patients discharging against medical advice accounts for approximately 1–2% of all hospital separations [1,2,3,4], although higher rates have been reported in certain medical subpopulations [2,4,5]. In Australia, between July 2011 and June 2013, 0.5% of all hospitalisations for non-Indigenous Australians were DAMA [6]. A recent study from the USA found that the rates for DAMA have increased over the past decade, making this an important public health issue [7] given these patients carry a risk of readmission and have increased morbidity and mortality [1,2,4,8]. Patients who DAMA also present clinical, ethical, financial and legal challenges for healthcare providers [1,2,4,9,10,11].

Predictors of DAMA in the adult population include younger age, male gender, substance abuse, mental health comorbidities, lower socio-economic class and rural residence [1,3,4,6,7,12]. A history of previous DAMA is also strongly associated with DAMA [5]. In Australian adults, Aboriginal ethnicity is the strongest predictor of DAMA, with 5% of all hospitalisations for Aboriginal and Torres Strait Islander peoples ending in DAMA, a rate 8 times that of the non-Aboriginal population [6,13].

There is limited data on DAMA in paediatric populations and in high income countries. The majority of research comes from low and middle-income countries (LAMI) in Africa and the Middle East, where rates of DAMA among paediatric patients vary both between and within nations, ranging from 1.5% to over 6% [11,14,15,16,17,18]. It has been suggested that DAMA rates in LAMI countries are two times higher than in high income countries [11], most likely related to financial issues and resource availability. Aboriginality is a risk factor for DAMA in children in Australia, where 1.3% of hospitalisations in Aboriginal children aged 4 years or less DAMA compared to 0.2% of non-Aboriginal children in the same age group, and 3.3% compared to 0.6% in children aged 5 to 14 years [6].

Research from paediatric emergency departments [19] and paediatric wards in both LAMI and high income countries has shown that predictors of DAMA in children are age (less than 2 years old or adolescent age-group), male gender, duration of hospital stay 48 hours or less, financial constraints, lack of health insurance and lower socio-economic status [20]. As with adult patients, children who are discharged by their parent or carer against medical advice from either hospital or an ED are at increased risk of readmission and increased morbidity due to deterioration of the medical condition at home, with associated increased costs of care [9,11,20]. Paediatric DAMA also creates a moral dilemma for paediatricians caught between respect for the parents’ autonomy and the desire to provide appropriate care for the child [9,11].

There is no published literature on rates or predictors of DAMA in the paediatric tertiary care setting. Identifying paediatric patients at high risk of DAMA allows for intervention in those at risk of adverse outcomes. Understanding the association between Aboriginality and DAMA is an important step in ensuring equitable, quality care is provided to Aboriginal children in Australia.

This study aims to identify the demographic and clinical characteristics of patients who discharge against medical advice (DAMA) from the Sydney Children’s Hospital’s Network in order to better understand this phenomenon in children seeking care and treatment in a tertiary paediatric setting.

## 2. Materials and Methods

This was a retrospective cross-sectional analysis of paediatric admissions to the Sydney Children’s Hospitals Network (SCHN), the largest paediatric healthcare entity in Australia. This network, split between two hospital sites (in Western and Eastern Sydney), provides healthcare primarily to the paediatric population of New South Wales. NSW is home to more Aboriginal people than any other state or territory, making up 3% of the general population and 5.4% of all children aged 0 to 14 years [21]. Data were encrypted and stored in the secure SCHN and the Sydney University Research Data Storage Facility with access provided only to members of the project team as defined in the ethics approval.

### 2.1. Ethical Issues

Approval was granted by the Aboriginal Health and Medical Research Council (AH&MRC), the SCHN Human Research Ethics Committee and the governance committees at both Sydney Children’s Hospital and Children’s Hospital at Westmead. The study team acknowledges the diversity of Aboriginal peoples, including their different languages, cultures, histories and perspectives. Aboriginal and/or Torres Strait Islander people are referred to as “Aboriginal”, in recognition that Aboriginal people are the original inhabitants of NSW [22]. This study was conducted in collaboration with the SCHN’s Aboriginal Health Management Advisor with the aim of considering the lived experience of Aboriginal peoples in data analysis and interpretation.

### 2.2. Participants

All inpatient admissions to SCHN between 1 January 2011 and 31 December 2015 were included whether they were admitted from the Emergency Departments, outpatients, or directly to the ward. Non-admitted patients presenting to the emergency and outpatient departments were excluded.

### 2.3. Patient and Admission Characteristics

Demographic and clinical characteristics were extracted from the Citrix Powerchart Electronic Medical Records database. “Mode of separation” data were used to identify patients who DAMA. All patients with other modes of separation (discharged by hospital, transferred to another hospital or facility, deceased or other) were classified as “non-DAMA” patients/admissions. Demographic characteristics extracted were age, sex, Aboriginality, postcode, suburb and local health district. Aboriginality included “Aboriginal but not Torres Strait Islander”, “Torres Strait Islander but not Aboriginal” and “Aboriginal and Torres Strait Islander”. Postcodes were used to determine socioeconomic status and remoteness of residence (rurality). The Socio-Economic Indexes of Areas (SEIFA) Index of Relative Socioeconomic Disadvantage (IRSD) was used as a measure of socioeconomic status. This is a general socioeconomic index that summarises a range of information about social and economic conditions of people and households within the area. It includes only measures of relative disadvantage. A low index score indicates a relatively greater disadvantage. The 2011 IRSD was obtained from the Australian Bureau of Statistics (ABS) and was matched to patient data by postcode and suburb name. Socioeconomic disadvantage was categorised into “low”, “medium” and “high”. Remoteness was measured using the Accessibility/Remoteness Index of Australia (ARIA+), which is a nationally recognised consistent measure of geographic remoteness. It was obtained from the ABS by Statistical Area Level 1 (SA1), converted to postcode and matched via postcode and suburb name. Remoteness was categorised into “major cities of Australia”, “inner regional Australia”, “outer regional Australia”, “remote Australia” and “very remote Australia”.

Clinical characteristics extracted were admitting hospital (hospital site 1 or hospital site 2), urgency of admission, diagnosis and previous DAMA within the 5-year period of the study. International Classification of Diseases—10th Revision (ICD-10) codes were used to determine a patient’s diagnosis category. They were divided into broad categories based on the first letter of the ICD-10 code, for example, infection (ICD code A or B), neoplasm (ICD code C or D) and mental health or behavioural (ICD code F). ICD-10 diagnosis codes were also used to identify potentially avoidable hospitalisations. These are diagnoses that might be influenced by access to primary health care as well as social measures such as housing, childcare and income support [23].

### 2.4. Statistical Analysis

We analysed patient demographics using the number of patients, counting only unique medical record numbers; and analysed clinical characteristics using the number of admissions, including multiple admissions per patient. We tested for significant differences in the rates of DAMA for each individual demographic and clinical characteristic using a chi-squared test. We then examined the independence of associations using Bayesian multivariable logistic regression to determine the predictors of DAMA in any given admission [24]. We present results from the regression analysis using odds ratios (OR) and 95% highest-probability density credible intervals. A *p*-value of less than 0.05 was considered significant.

## 3. Results

### 3.1. Demographic and Clinical Characteristics

There were a total of 246,359 admissions for 124,757 individual patients over the 5-year period. A total of 1871 admissions (0.8%) and 1730 unique patients (1.4%) DAMA. Of the non-DAMA admissions, 239,678 (97.3%) were discharged home, 4016 (1.6%) were transferred to another hospital, 543 (0.2%) died and 305 (0.1%) had “other” modes of separation such as transfer to another facility.

Of all children admitted, there were more males than females (58% vs. 42%) and the majority were young children in the 2–6-year age group. There were 2.9% who identified as “Aboriginal and/or Torres Strait Islander”, 96.6% who did not identify as Aboriginal and 0.6% had unknown Aboriginality. The majority of patients resided in major cities (90%), followed by inner regional (7.1%), outer regional (1.9%), remote (0.2%) and very remote (0.1%) areas. Most patients were from higher socioeconomic status areas (54.9% high and 40% medium), with only a small proportion (5.1%) from the most disadvantaged areas (Table 1).

Hospital site 1 in Western Sydney had more admissions than hospital site 2 in Eastern Sydney (61.5% vs. 38.5%). Hospital sites 1 and 2 were similar in their distribution of gender, rurality and urgency of admission. The proportion of Aboriginal patients admitted to site 2 was higher than site 1 (3.34% vs. 2.96%). Site 2 had a higher proportion of younger children (age 0–6 years) and site 1 had a higher proportion of older children (8–18 years). Patients admitted to site 1 were more likely to be from lower socioeconomic status areas compared with site 2; site 1 had a higher proportion of patients in the “low” (7.3% vs. 2.3%) and “medium” (47.7% vs. 31.9%), and less in the “high” IRSD category (45.0% vs. 65.7%) (Table 2).

Planned admissions were more common than emergency admissions (50.8% vs. 45%) and the most common admitting diagnostic categories were injury/poisoning (12.9%), respiratory (11.6%), neoplasm/blood/immune (10.0%) and congenital and chromosomal (9.1%) conditions. Potentially avoidable hospitalisations, such as respiratory tract infections and vaccine preventable diseases, accounted for 19.9% of all admissions. Most patients had a short length of stay; 68% of admissions (166,745) were less than 24 hours in duration. 

The univariate analysis found DAMA to not be independent from the following demographic variables: hospital site (*p* < 0.01), Aboriginality (*p* < 0.01), age group (*p* < 0.01) and IRSD (*p* < 0.01); as well as the following clinical variables: emergency admissions (*p* < 0.01) and ICD-10 diagnosis code (*p* < 0.01). A linear model found no effect of DAMA on time until next admission (*p* < 0.05).

### 3.2. Predictors of Discharge Against Medical Advice (DAMA) 

Predictors of DAMA were hospital site 1, emergency admissions, a mental health or behavioural diagnosis, Aboriginality, a “digestive” diagnosis and previous DAMA. Age, length of stay, socioeconomic status and remoteness were not significant predictors of DAMA in the final logistic regression model (Table 3). The hospital site 1 was the strongest predictor of DAMA with a patient admitted to hospital site 1 almost 5 times more likely to DAMA than a patient admitted to hospital site 2 (OR 4.8, CI 4.2–5.7; *p* < 0.01). Patients with a primary mental health or behavioural diagnosis were 3 times more likely to DAMA than the most populous “infection” reference category (OR 3.3, CI 2.2–4.8; *p* < 0.01). Having a primary diagnosis of the “digestive system” also increased the likelihood of DAMA (OR 1.5, CI 1.1–2.0; *p* = 0.04). Aboriginal patients were significantly more likely to DAMA (OR 1.6, CI 1.3–2.1; *p* < 0.01). Planned admissions were less likely to DAMA than emergency admissions (OR 0.69, CI 0.61–0.79; *p* < 0.01). Patients who had DAMA previously within the study time frame were also more likely to DAMA (OR 2.1, CI 1.2–3.2; *p* = 0.05).

## 4. Discussion

This is the first study describing rates and characteristics of inpatients that DAMA in a paediatric tertiary care setting. The overall rate of DAMA seen in this cohort was lower at 0.8% of all admissions relative to the 1–2% reported in the adult literature [1,2,3,4]. It is also lower than the rates reported in paediatric populations globally, which vary between 1.5% to over 6% [11,14,15,16,17,18]. Direct comparisons with other studies are difficult as they differ considerably in study setting and patient cohort. Much of the DAMA research in children comes from countries such as Nigeria, Iran and Oman, or are set in the Emergency Departments of general hospitals. There are no published studies of DAMA from a tertiary referral paediatric centre.

Predictors of DAMA seen in our study were hospital site, a mental health/behavioural diagnosis, emergency admission, Aboriginality, previous DAMA and a “digestive” diagnosis. The increased DAMA rates in Aboriginal as compared with non-Aboriginal children in Australia are reflected in other Australian data where DAMA has been documented at around 6 times the rate [6]. We did not find age (less than 2 years or adolescence), duration of hospital stay or lower socio-economic status significant for predicting DAMA, although these have been reported in the literature [20,25]. It is conceivable that duration of hospital stay and socio-economic status are less predictive in the Australian context as the patient is not required to pay for their hospital stay due to a government funded hospital service, although age can be a predictor even in Australia [6].

Hospital characteristics have been described as impacting rates of DAMA, with a study of 995 acute care hospitals in the USA finding urban location and medium or large size hospitals to be associated with higher risk of DAMA and teaching hospital status with lower risk of DAMA [3]. Our study sites were both teaching hospitals and this may account for the lower DAMA, but it is unclear from the data why the rates of DAMA were so different between the two sites. We know that the hospital with higher DAMA rates is larger and covers a population that is more culturally and linguistically diverse. As such, we can speculate that cultural and linguistic diversity may have an impact on DAMA due to increased barriers to healthcare such as communication difficulties.

Planned admissions were less likely than emergency admissions to DAMA in our study. We hypothesise that this is related to patients and their families’ expectations and capacity to plan, for example, for sibling care and leave from work, when they come to hospital. The length of stay of a planned admission is generally controlled and communicated to the family prior to admission, and this predictability is likely to influence a patient’s decision to DAMA from hospital.

Mental health diagnoses are known risk factors in the adult population [1] and therefore it was not surprising that this was found to be a predictor in the paediatric population. “Digestive” diagnoses have not been widely reported as a risk factor for DAMA, but one study did identify it as a predictor for DAMA in emergency department presentations [20].

Consistent with the literature, we found that having previously DAMA within the study period was a predictor for future DAMA events [5,12]. A large, population-based study of all acute hospital admissions among adults in a province in Canada over 19 years found that 19.1% of people who ever DAMA did so more than once, accounting for 41.2% of all DAMA events. Although they offer no explanation for this finding, it is noted that the likelihood that a given patient would DAMA did not change as that person aged, indicating that whatever predisposes people to DAMA does not change over time. More research is required to understand this, but identification of previous DAMA may allow clinical teams to be alert to the increased risk and to intervene with additional support during hospitalisation to limit the chance of repeated DAMA [5].

Aboriginality is known to be a strong predictor of DAMA in both Australian adults and children [12]. A significantly greater proportion (2%) of Aboriginal children in this cohort DAMA as compared to non-Aboriginal children (1.4%). Aboriginality is a known risk factor for DAMA in children in Australia across all age groups [6]. DAMA is considered an indirect measure of the cultural competency of health care services and, in the case of Aboriginal Australians, may reflect suboptimal responsiveness of hospitals to Aboriginal needs and poorer quality of care [13]. This has been well-documented in Australian hospitals and has been termed “institutional racism”, [26]. The provision of culturally appropriate care for Aboriginal patients is directly linked to health outcomes [27]. The level of trust in the health system, staff attitudes, hospital policies and historical issues such as racism, foster a lack of cultural safety within health services and may explain the increased likelihood of DAMA [6,19]. Understanding the reasons why Aboriginal patients discharge against medical advice more frequently than non-Aboriginal patients requires high quality Aboriginal-led research and the formulation of appropriate strategies to address the problem, especially since Aboriginal health outcomes are significantly worse than those of their non-Aboriginal counterparts [28].

Although this study could not assess patient reasons for DAMA, the literature cites personal or family circumstances, financial constraints, feeling well and dissatisfaction with treatment as common reasons [2,3,15]. Parents whose children DAMA report a perception that the child is well, the inconvenience of the hospital stay, financial constraints, dissatisfaction of care received and preference for alternative or traditional treatments [9,11,14]. In many LAMI studies, the cost of health care, the cultural importance of rituals and seeking traditional therapies in preference to modern treatment are reasons for DAMA [11,15].

The main limitation of this study was that the data used was extracted from electronic medical records, which relies upon administrative and medical staff entering data accurately. Of particular importance is the probable under-documentation of Aboriginal status. This is likely given that 2.9% of the cohort were Aboriginal, whereas 5.4% of all children and adolescents in NSW are Aboriginal [21]. Under-identification of Aboriginality in hospital systems may be due to fear of racist treatment and the historical practice of removal of children during their hospital stays. It has been well documented that there is under-identification in Aboriginality in administrative health data in Australia [29]. Data linkage can be used to more accurately assign Aboriginal ethnicity; however, this was out of the scope of this study [29].

## 5. Conclusions

The rate of DAMA and the clinical and demographic factors that predict it have not previously been reported in the paediatric population in a tertiary setting. Clinician awareness of patient groups at risk for DAMA may allow intervention and support to prevent known adverse outcomes. Understanding the specific populations at a higher risk of DAMA may provide insights into aspects of health services that need to be addressed to improve quality of care for all patients. Further research is required to explore the factors leading to DAMA, as well as effective strategies to address it at a practice and policy level. Particular attention to improving the cultural competency of health services may increase access and acceptability for Aboriginal populations, which may in turn improve the disparities in health outcomes.

## Figures and Tables

**Table 1:** Demographic characteristics (by number of patients).

Demographic Characteristics	Total n (%)	DAMA n (DAMA %)	*χ*^2^, df, *p*-Value
Overall	124,757 (100%)	1730 (1.4%)	
**Gender**			*χ*^2^ = 0.44
Male	72,304 (58%)	989 (1.4%)	df = 1
Female	52,453 (42%)	741 (1.4%)	*p* = 0.50
**Age group**			
Neonate (0–28 days)	5823 (4.7%)	87 (1.5%)	*χ*^2^ = 30.0
Infant (29 days–23 months)	36,608 (29.3%)	493 (1.3%)	df = 5
Young Child (24 month–6 years)	39,162 (31.4%)	498 (1.3%)	*p* < 0.01
Child (7–12 years)	28,559 (22.9%)	386 (1.4%)	
Adolescent (13–17 years)	14,451 (11.6%)	260 (1.8%)	
Adult (18 years or more)	154 (0.1%)	6 (3.9%)	
**Aboriginality**			
Aboriginal	3565 (2.9%)	76 (2.1%)	*χ*^2^ = 15.7
Not Aboriginal	120,474 (96.6%)	1647(1.4%)	df = 2
Unknown	718 (0.6%)	7 (1.0%)	*p* < 0.01
**Hospital site**			*χ*^2^ = 553
Hospital site 1	76,693 (61.5%)	1536 (2%)	df = 1
Hospital site 2	48,064 (38.5%)	194 (0.4%)	*p* < 0.01
IRSD Category *			
Low 400–850	6350 (5.1%)	121 (1.9%)	*χ*^2^ = 42.7
Med 851–1000	49,492 (40%)	775 (1.6%)	df = 2
High 1001–1150	67,901 (54%)	810 (1.2%)	*p* < 0.01
Unmatched (not incl. in test)	1014 (0.8%)	24 (2.4%)	
**Remoteness**			
Major cities	112,204 (90%)	1531 (1.4%)	*χ*^2^ =2.84
Inner regional	8885 (7.1 %)	136 (1.5%)	df = 4
Outer regional	2388 (1.9%)	34 (1.4%)	*p* = 0.72
Remote	256 (0.2%)	3 (1.2%)	
Very Remote	101 (0.1%)	2 (2.0%)	
Unmatched (not incl. in test)	923 (0.7%)	24 (2.6%)	

* Index of Relative Socioeconomic Disadvantage (IRSD) was used as a measure of socioeconomic status. This is a general socioeconomic index that summarises a range of information about social and economic conditions of people and households within the area. It includes only measures of relative disadvantage. A low index score indicates a relatively greater disadvantage.

**Table 2:** Clinical characteristics (by number of admissions).

Clinical Characteristics	Total n (%)	DAMA n (DAMA %)	*χ*^2^, df, *p*-Value
Total Admissions	246,359 (100%)	1817 (0.7%)	
**Urgency**			
Emergency	111,707 (45.3%)	969 (0.9%)	*χ*^2^ = 53.5
Planned	125,176 (50.8%)	768 (0.6%)	df = 2
Other	9476 (3.8%)	80 (0.8%)	*p* < 0.01
**Diagnosis (ICD-10)**			
A,B—Infection	10,590 (4.3%)	77 (0.7%)	*χ*^2^ = 184.6
C,D—Neoplasm/Blood/Immune	24,753 (10.0%)	158 (0.6%)	df = 17
E—Endocrine/Nutritional/Metabolic	8168 (3.3%)	83 (1.0%)	*p* < 0.01
F—Mental, Behavioural	2593 (1.1%)	58 (2.2%)	
G—Nervous system	14,118 (5.7%)	80 (0.6%)	
H—Eye, Ear, Adnexa	7520 (3.1%)	61 (0.8%)	
I—Circulatory	2646 (1.1%)	20 (0.8%)	
J—Respiratory	28,579 (11.6%)	226 (0.8%)	
K—Digestive	19,762 (8.0%)	170 (0.9%)	
L—Skin	7028 (2.9%)	51 (0.7%)	
M—Musculoskeletal	9531 (3.9%)	74 (0.8%)	
N—Genitourinary	9607 (3.9%)	51 (0.5%)	
O—Pregnancy & Childbirth	5 (0.002%)	0 (0%)	
P—Perinatal	2490 (1.0%)	17 (0.7%)	
Q—Congenital & chromosomal	22,478 (9.1%)	133 (0.6%)	
R—Other symptoms	21,093 (8.6%)	216 (1.0%)	
S,T—Injury/poisoning	31,718 (12.9%)	225 (0.7%)	
Z—Other diagnosis	21,684 (8.8%)	92 (0.4%)	
Missing/Unmatched	1996 (0.8%)	25 (1.3%)	
**Potentially Preventable Hosp.**			*χ*^2^ = 2.4
No	197,324 (80.1%)	1429 (0.7%)	df = 1
Yes	49,035 (19.9%)	388 (0.8%)	*p* = 0.12

**Table 3:** Predictors of DAMA per admission (Bayesian multivariable logistic regression).

Characteristic		OR	(95% CI)	*p*-Value
**Hospital**	**Hospital 1**	**4.77**	**(4.24** **–5.69)**	**<0.01**
	Hospital 2	1.00		
**Diagnosis (ICD10)**	A,B—Infection	1.00		
	C,D—Neoplasm, Blood, Immune	1.18	(0.85–1.56)	0.47
	E—Endocrine, Nutritional, Metabolic	1.55	(1.06–2.12)	0.09
	**F** **—Mental, Behavioural**	**3.35**	**(2.20** **–4.82)**	**<0.01**
	G—Nervous system	1.08	(0.80–1.77)	0.62
	H—Eye, Ear, Adnexa	1.35	(0.98–2.02)	0.20
	I—Circulatory	1.30	(0.74–2.13)	0.53
	J—Respiratory	1.15	(0.86–1.44)	0.52
	**K** **—Digestive**	**1.51**	**(1.12** **–2.02)**	**0.04**
	L—Skin	1.18	(0.79–1.62)	0.54
	M—Musculoskeletal	1.23	(0.84–1.64)	0.49
	N—Genitourinary	0.93	(0.61–1.26)	0.70
	P—Perinatal	1.39	(0.81–2.18)	0.38
	Q—Congenital, Chromosomal	1.12	(0.82–1.53)	0.64
	R—Other Symptoms	1.45	(1.10–1.90)	0.07
	S,T—Injury, Poisoning	1.09	(0.77–1.46)	0.75
	Z—Other	0.80	(0.55–1.13)	0.45
**Urgency of**	Emergency	1.00		reference
**Admission**	**Planned**	**0.69**	**(0.61** **–0.79)**	**<0.01**
**Aboriginality**	Not Aboriginal	1.00		reference
	**Aboriginal**	**1.63**	**(1.29** **–2.13)**	**<0.01**
**Previous DAMA**	No Prev. DAMA	1.00		reference
	**Prev. DAMA**	**2.06**	**(1.18** **–3.17)**	**0.05**
**Remoteness**	Metropolitan	1.00		reference
	Inner Regional Australia	1.00	(0.84–1.20)	0.85
	Outer Regional Australia	0.95	(0.62–1.24)	0.67
	Remote Australia	0.44	(0.02–0.96)	0.35
	Very Remote Australia	1.23	(0.11–3.81)	0.82
**IRSD**	Social disadvantage (numeric)	1.00	(1.00 – 1.00)	0.52
**Potentially**	Not Preventable	1.00		reference
**Preventable Hosp.**	Preventable	0.95	(0.79–1.13)	0.63
**Age**	Age (numeric)	1.00	(0.99–1.01)	0.75
**Gender**	Male	1		reference
	Female	0.97	(0.88–1.10)	0.77
